# Integrative analysis of cell adhesion molecules in glioblastoma identified prostaglandin F2 receptor inhibitor (PTGFRN) as an essential gene

**DOI:** 10.1186/s12885-022-09682-2

**Published:** 2022-06-11

**Authors:** Uchurappa Mala, Tapan Kumar Baral, Kumaravel Somasundaram

**Affiliations:** Department of Microbiology and Cell Biology, Indian Institute of Science, Bangalore, 560012 India

**Keywords:** Glioblastoma, CAM, PTGFRN, GSC, Growth, Migration, miR-137

## Abstract

**Background:**

Glioblastoma (GBM) is the most common primary malignant brain tumor in adults exhibiting infiltration into surrounding tissues, recurrence, and resistance to therapy. GBM infiltration is accomplished by many deregulated factors such as cell adhesion molecules (CAMs), which are membrane proteins that participate in cell-cell and cell-ECM interactions to regulate survival, proliferation, migration, and stemness.

**Methods:**

A comprehensive bioinformatics analysis of CAMs (*n =* 518) in multiple available datasets revealed genetic and epigenetic alterations among CAMs in GBM. Univariate Cox regression analysis using TCGA dataset identified 127 CAMs to be significantly correlated with survival. The poor prognostic indicator PTGFRN was chosen to study its role in glioma. Silencing of PTGFRN in glioma cell lines was achieved by the stable expression of short hairpin RNA (shRNA) against the PTGFRN gene. PTGFRN was silenced and performed cell growth, migration, invasion, cell cycle, and apoptosis assays. Neurosphere and limiting dilution assays were also performed after silencing of PTGFRN in GSCs.

**Results:**

Among the differentially regulated CAMs (*n =* 181, 34.9%), major proportion of them were found to be regulated by miRNAs (*n =* 95, 49.7%) followed by DNA methylation (*n =* 32, 16.7%), and gene copy number variation (*n =* 12, 6.2%). We found that PTGFRN to be upregulated in GBM tumor samples and cell lines with a significant poor prognostic correlation with patient survival. Silencing PTGFRN diminished cell growth, colony formation, anchorage-independent growth, migration, and invasion and led to cell cycle arrest and induction of apoptosis. At the mechanistic level, silencing of PTGFRN reduced pro-proliferative and promigratory signaling pathways such as ERK, AKT, and mTOR. PTGFRN upregulation was found to be due to the loss of its promoter methylation and downregulation of miR-137 in GBM. PTGFRN was also found to be higher in glioma stem-like cells (GSCs) than the matched differentiated glioma cells (DGCs) and is required for GSC growth and survival. Silencing of PTGFRN in GSCs reduced transcript levels of reprogramming factors (Olig2, Pou3f2, Sall2, and Sox2).

**Conclusion:**

In this study, we provide a comprehensive overview of the differential regulation of CAMs and the probable causes for their deregulation in GBM. We also establish an oncogenic role of PTGFRN and its regulation by miR-137 in GBM, thus signifying it as a potential therapeutic target.

**Supplementary Information:**

The online version contains supplementary material available at 10.1186/s12885-022-09682-2.

## Background

Glioblastoma (GBM) is the most common primary malignant tumor of brain neoplasia in adults, and GBM patients have the worst prognosis with a median survival of around 1 year [1]. GBM cells infiltrate into the brain parenchyma and are responsible for the complications encountered in surgery, therapy, and the lethality of the disease [2].

Adhesion of cells to a substratum, extracellular matrix (ECM), and to one another is primarily accomplished by a family of surface proteins called cell adhesion molecules (CAMs), which are involved in the regulation of normal development and pathology of diseases including cancer [3]. Initially, CAMs were depicted as tumor suppressors; however, many reports emphasized their oncogenic functions in several cancers, including glioma [4, 5].

Prostaglandin F2 receptor inhibitor (PTGFRN) is a type I (single pass) transmembrane Ig superfamily CAM, which was shown to be upregulated in several cancers, including glioma [6–8]. PTGFRN exhibits gene fusion (PTGFRN-NOTCH2) in colorectal cancer [9] and point mutations in small cell lung cancer [10]. It interacts with tetraspanins (CD9 and CD81) [11, 12], integrins [11], Ezrin-Radixin-Moesin (ERM) proteins [13], and γ-secretase to regulate cell adhesion and migration [14, 15]. It was also found to be involved in adipocyte maturation [16], muscle regeneration [17], tumor angiogenesis [18], metastasis [7], inhibition of follicle-stimulating hormone (FSH) and luteinizing hormone (LH) secretion [19], and plasmodium infection [20]. Recently, PTGFRN was shown to be overexpressed in GBM, promoting cell growth and resistance to radiation via PI3K-AKT signaling [21]. However, its role in migration, invasion, and regulation of its expression in GBM is unknown.

Though several reports emphasize the importance of individual CAMs in GBM initiation and progression, no attempt has been made to study them comprehensively. In the present study, we analyzed 518 CAMs for their transcriptional changes in GBM using multiple datasets and explored the probable causes for the deregulation. Further, we also provide experimental evidence to establish the pro-proliferative, promigratory function, and regulation of PTGFRN, an upregulated CAM, in GBM.

## Materials and methods

### Compilation of CAMs

A list of manually curated 518 CAMs **(Supplementary Table** **1****)** was prepared from various sources such as Entrez query ‘CAMs and homo sapiens’, and gene ontology (GO) term annotations related to cell adhesion was used for the analysis in this study.

### Differential expression analysis

The gene expression data for GBM was downloaded from TCGA, REMBRANDT, GSE7696, and GSE22866 datasets. The differential expression was calculated by subtracting the average value of control samples from the average of GBM samples. Statistical significance was tested using the Wilcoxon-Mann-Whitney test. Genes showing fold change ≤ − 0.58 or ≥ 0.58 and significant *p*-value (*p*-value≤0.05; t-test: Wilcoxon-Mann-Whitney test and additional Benjamini/Hochberg FDR correction was applied) were considered as differentially expressed.

### Immunohistochemistry analysis

For PTGFRN protein level validation, we utilized the immunohistochemical data derived from the Human Protein Atlas (www.proteinatlas.org). The antibody used for this analysis is from Sigma-Aldrich (Cat# HPA017074; Rabbit polyclonal antibody). The IHC staining intensity was scored on a scale of 0-3 (0-no staining, 1+ -weak staining, 2+ -moderate staining, 3+-strong staining) with 1+ and above was considered as positive. The cells showing percent tumor cell positivity values >25, <25%, and none were considered.

### Survival analysis

All CAMs were subjected to Univariate Cox regression analysis using TCGA Agilent dataset and SPSS software. GraphPad Prism software 5.0 was used for the Kaplan-Meier survival analysis.

### Copy number variation

Copy number variation of CAMs was analyzed using data from cBioPortal (http://www.cbioportal.org/) and calculated the percentage of samples in which a particular CAM was amplified or deleted.

### Methylation data analysis

The list of CpGs corresponding to differentially expressed genes was fetched from the TCGA DNA Methylation dataset (Illumina Infinium Human DNA methylation 450 K array). The CpG probes present in all parts of the gene were considered for this analysis. The data for control samples were taken from GSE79122. For each probe, the differential beta value (methylation value) was calculated by subtracting the average beta value of the control from the average beta value of the GBM. A difference of >0.3 absolute beta value was applied to identify differentially methylated probes. The Wilcoxon-Mann-Whitney test was used to calculate statistical significance.

### MicroRNA

The differentially regulated CAMs were used as an input for miRwalk. The miRNAs which were predicted by a minimum of seven or more algorithms to target the CAMs were taken for further analysis. The miRNAs that were reciprocally regulated with respect to their target CAMs were considered as miRNA and CAM pairs.

### Cell lines, normal brain tissues, and plasmids

Glioma (U373, T98G, U251, U87, LN229, U343, LN18, A172, and U138), Immortalized human astrocytes (IHA and SVG), and 293T cell lines were cultured in DMEM (Sigma-Aldrich, #D5648) supplemented with 10% Fetal Bovine Serum (FBS), Penicillin, and Streptomycin. U251, U87, U373, T98G, and 293T were bought from ECACC. LN229 and IHA were gifted by late Dr. Abhijit Guha (University of Toronto, Canada). Patient tumor-derived primary GSC lines MGG8, MGG6, and MGG4 were procured from Dr. Wakimoto H. (Massachusetts General Hospital, Boston, USA), and 1035 was obtained from Dr. Santosh Kesari (University of California, San Diego, USA) and were cultured as neurospheres.

Non-tumorous control brain tissue samples (N1-N5) were procured from patients with intractable epilepsy during surgery at the National Institute of Mental Health and Sciences (NIMHANS), Bangalore, India. The tissue samples were obtained with written consent from all patients before using them in the current study. The study has been approved by the ethics committee of the NIMHANS and Indian Institute of Science (IISc). In the present study, control brain tissue samples were used for the isolation of RNA and to measure PTGFRN transcript levels.

PTGFRN shRNAs TRCN00000057448 to TRCN00000057452 (Sigma-Aldrich) were used for silencing PTGFRN, and pcDNA3.2/V5-mmu-miR-137 (Addgene) was used for overexpression of miR-137. The luciferase reporter construct pmiR-GLO-3’UTR of PTGFRN was a kind gift from Dr. Markus Stoffel (Institute of Molecular Health Sciences, ETH Zurich, Switzerland).

### Lentivirus preparation and transduction

293T cells were transfected with shRNA (4 μg) along with the helper plasmids pSPAX and pMD2.G (3:1) using Lipofectamine 2000 (Invitrogen, #11668019) transfection reagent in a 60 mm dish. The media was changed after 5 hours of transfection, and supernatant, which contains virus particles, was collected after 60 h of transfection. The virus suspension was used to infect glioma cells in the presence of 8 μg/ml of Polybrene (Sigma, #107689).

### cDNA conversion and qPCR

The total RNA was isolated by using Trizol (Sigma-Aldrich, #T9424) method. RNA was converted to cDNA by using cDNA conversion kit (Life Technologies, #4368813). Subsequently, RT-qPCR was performed, and fold change was calculated by the ΔΔct method.

### Western blotting

The RIPA buffer was used to lyse the cells, and the supernatant was collected. Protein was quantified using Bradford’s reagent, and the required amount of protein was resolved in the SDS-PAGE gel, and Western blotting was performed. The primary antibodies anti-PTGFRN (#ab97567, Abcam), anti-phospho-ERK1/2 (#9101, CST), anti-ERK (#9102, CST), anti-phospho-AKT (#9271, CST), anti-AKT (#4691, CST), anti-phospho-p70S6 (#9208, CST), anti-p70S6 (#2708, CST), anti-phospho-4EBP1 (#9456, CST), anti-4EBP1 (#9452, CST), and anti-β-actin (#A3854, Sigma-Aldrich) were used in this study.

### Proliferation assay

The cell viability was assessed by trypan blue assay. Briefly, the cells expressing either control non-targeting shRNA (shNT) or shRNA against PTGFRN (shPTGFRN) were plated in a six-well plate with 10^4^ cells per well. Cell viability was checked every 3^rd^ day using the Vi-cell counter (#383722, Beckman Coulter), and normalization was done using the reading of day-1 for each condition. Statistical analysis was done using the Student’s t-test.

### Colony suppression assay

The cells stably expressing shRNAs were counted and plated in a six-well plate in triplicates at the seeding density of 10^3^ cells/well and grown for 2-3 weeks replacing the media every 2-3 days. Colonies were fixed using chilled methanol overnight, followed by staining with crystal violet (0.05% w/v) for 30 min. Colonies were quantified by counting using ImageJ software. The statistical significance was calculated by the Student’s t-test.

### Soft agar colony formation assay

In the soft agar assay, cells were counted, and 10^4^ cells/well in 1.2 ml of 0.4% low melting agarose (#214230, BD Biosciences) was plated in a six-well plate containing 0.6% agarose base layer. Each condition was plated in triplicates. After 2-3 weeks, images were taken, and quantification was performed. Statistical analysis was done using the Student’s t-test.

### Migration and invasion assays

Trans-well matrigel invasion assay is an in-vitro method to assess the ability of cells to degrade extracellular matrix proteins in response to a stimulus. Migration of cancer cells was assayed in 24 well Boyden chamber with 8 μm pore size polycarbonate membrane (BD Biosciences, San Diego, USA). For invasion assay, the membranes pre-coated with matrigel (a mix of extracellular matrix proteins) were used (BD Biosciences, Sandiego, USA). The matrigel layer serves as a reconstituted basement membrane that occludes the pores. Cells (5 × 10^4^) were resuspended in 500 μl serum-free medium and placed in the upper chamber, and the lower chamber was filled with 600 μl medium with 10% FBS (serving as a chemo-attractant). Cells were incubated for 22 hours, and after incubation, the cells remaining on the upper surface of the membrane were removed by wiping with a wet cotton bud. The cells on the lower surface of the membrane were fixed using chilled methanol and stained with 0.05% crystal violet and counted under the light microscope. The statistical significance was calculated by the Student’s t-test.

### Cell cycle analysis by flow cytometry

For analyzing the percentage of cells in different phases of the cell cycle, both the floating and adherent cells were used. The adherent cells were washed with PBS and harvested by trypsinization. The single-cell suspension was pelleted down and resuspended in 300 μl of PBS and fixed with chilled 100% ethanol (700 μl) by adding drop by drop while gentle vortexing. Cells were fixed by incubating them at −20 °C overnight. Incubation was followed by re-pelleting cells and complete removal of ethanol by two PBS washes followed by treatment with RNase A (10 μg/ml) for 2-3 h at 37 °C. Cells were stained with propidium iodide (PI), 10 μg/ml, and subjected to flow cytometry using FACS-VERSE instrument (BD Biosciences) to assay the effects on cell cycle profile. The statistical significance was calculated by the Student’s t-test.

### Annexin V-FITC (fluorescein isothiocyanate) and PI staining

Annexin V-FITC/PI double staining was utilized to quantify apoptosis in each condition. The apoptotic cells were determined under different conditions using flow cytometry-based analyses using Annexin V-FITC Apoptosis kit from Bio-vision (K-101) following the manufacturer’s instructions. The percentage of the healthy population, early/late apoptotic population, and necrotic population were determined from each condition, and the percentage of apoptotic cells was plotted. The statistical significance was calculated by the Student’s t-test.

### TaqMan advanced miRNA assay for measuring miR-137

To measure the miR-137 levels in IHA and GBM cell lines, we extracted the total small RNA from cell lines using Trizol lysis followed by column-based RNA purification method (miRNeasy Mini kit, #1038703; Qiagen). To synthesize cDNA from small RNA, TaqMan advanced miRNA cDNA synthesis kit was used (#A25576; Thermo Fisher Scientific, USA). To quantify the specific mature miR-137, TaqMan advanced miRNA assay (has-miR-137; Assay ID: 477904_mir; Thermo Fisher Scientific, USA) was performed using RT-qPCR method and miR-191 was used as an internal control.

### Luciferase reporter assay

293T cells were co-transfected with 1000 ng of pcDNA3.2/V5 vector or pcDNA3.2/V5-miR-137 overexpression plasmid along with 100 ng of pmiR-GLO-3’UTR of PTGFRN luciferase reporter plasmid in a 12 well-plate. The pmiR-GLO-3’UTR of PTGFRN construct contained ~1 kb insert DNA of 3’UTR of mouse PTGFRN and which covers one binding site for miR-137 in the middle of the insert [19]. After 48 hours of transfection, cells were harvested and assayed for luciferase activity using dual luciferase assay system (Promega). Firefly luciferase activity was normalized with Renilla luciferase activity, an internal control.

### GSCs and DGCs

MGG8, MGG6, MGG4, and 1035 GSCs were cultured as neurospheres in Neurobasal medium (#21103049, Invitrogen) supplemented with EGF (20 ng/ml; #236-EG-200, R&D systems), bFGF (20 ng/ml; #100-18B, Peprotech), 0.5x N-2 (#17502-048, Invitrogen), 1x B27 supplement (#17504-044, Invitrogen), 2 μg/ml Heparin (#H3149, Sigma) in ulta-low attachment dishes. For differentiation of GSCs to get DGCs, GSCs were transferred to 10% serum containing DMEM media in regular adherent plates and cultured for 7 days. The statistical significance was calculated by the Student’s t-test.

### Neurosphere assay and limiting dilution assay

GSCs were infected with lentivirus expressing either shNT or shPTGFRN. After 48 h of infection, the sphere aggregates formed were dissociated into single cells, counted, and plated at a density of 10^4^ cells/well in an ultra-low attachment six-well plates and cultured for 7 days. Fresh medium was replenished every 2-3 days. The number of spheres was counted after 7 days of plating, and graphs were plotted. The statistical significance was calculated by the Student’s t-test.

For limiting dilution assay, neurospheres were dissociated into single cells and counted. Cells were plated in an ultra-low attachment 96 well plates wherein a range of cells (1, 10, 25, 50, 75, and 100 cells/well) were plated into eight wells for each condition. Fresh medium was replenished every 2-3 days. After 7-10 days, wells forming the spheres were counted in control knockdown and gene knockdown conditions. A graph was plotted using ELDA (Extreme Limiting Dilution Analysis) software.

## Results

### Regulated CAMs in GBM

**Fig. 1:**
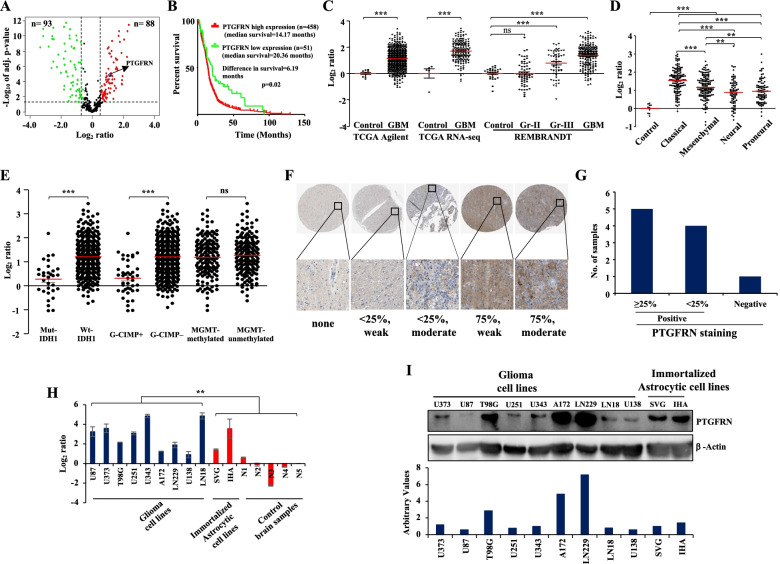
Transcriptional aberrations identified in CAMs in GBM predict the pro-tumorigenic potential of PTGFRN in GBM. **A** Volcano graph depicting upregulated (red), downregulated (green), and unregulated (black) CAMs in GBM samples (*n =* 572) as compared to control samples (*n =* 10). The horizontal line separates the CAMs having a significant difference in expression (*p*-value≤0.05). Vertical lines show the cut-off value ≤− 0.58 or ≥0.58 log_2_ ratio for classifying differentially regulated CAMs. **B** Kaplan-Meier curve shows the overall survival difference between PTGFRN-high and low transcript groups of GBM. Scatter plots show the transcript level of PTGFRN in GBM in - **C **TCGA Agilent, TCGA RNA-Seq, and REMBRANDT, **D** TCGA GBM subtypes: classical, mesenchymal, neural, and proneural, **E** mut-IDH1 and wt-IDH1, G-CIMP+ and G-CIMP–, and MGMT methylated and MGMT unmethylated groups. **F** Immunohistochemical (IHC) analysis for PTGFRN in GBM tissues, percent tumor cell positivity is indicated, **G** quantification shows percent tumor cell positivity in a given number of tissues samples. **H** Bar graph shows transcript level of PTGFRN in GBM cell lines and immortalized human astrocytes (SVG and IHA), and control brain samples as measured by RT-qPCR. **I** Immunoblot shows protein level of PTGFRN in GBM cell lines and immortalized human astrocytes and β-Actin served as a loading control (required portion of the blot is shown after cropping from the whole blot for both the proteins). The normalized protein levels are shown in the bar diagram. The significance was tested using the Wilcoxon-Mann-Whitney test and the symbols are indicated as follows: (ns) not significant; (*) *p ≤* 0.05; (**) *p ≤* 0.01 and (***) *p ≤* 0.001

To elucidate the deregulation of CAMs in GBM, we manually curated a comprehensive list of 518 CAMs **(Supplementary Table** **1****)** from various sources [22, 23]. We performed an integrative bioinformatics analysis to find out the deregulated CAMs in GBM and the reasons for their deregulation **(Supplementary Fig.** **1****)**. To identify the transcriptional changes in CAMs in GBM, we analyzed the expression of CAMs in the TCGA dataset (*n =* 582). We found a total of 181 CAMs to be differentially regulated in GBM as compared to control samples. Among the deregulated CAMs, 88 were significantly upregulated, and 93 were significantly downregulated **(**Fig. 1A**)**. We found nearly an equal percentage (48 and 52%) of CAMs differentially expressed in GBM compared to control. We observed similar results in the REMBRANDT dataset **(Supplementary Fig.** **2****A)**, and this was also validated in GSE22866 and GSE7696 datasets. We observed that 40-79% of 181 CAMs to be differentially regulated similarly in these datasets **(Supplementary Fig.** **2****B, C, Supplementary Table** **2****)**. Thus, we identified differentially regulated CAMs in GBM, indicating that CAMs might be playing both oncogenic and tumor suppressor functions in GBM.

We examined the possible mechanisms behind the differential regulation of CAMs in GBM. The copy number variation analysis in TCGA data showed that out of the 88 upregulated CAMs, five were amplified, and among 93 downregulated CAMs, seven were deleted in more than 1% of tumors **(Supplementary Fig.** **3****A, Supplementary Table** **3****)**. Thus, 5.68% of upregulated CAMs were found to be amplified, whereas 7.52% of downregulated CAMs were found to be deleted at their gene loci. Further, we examined the role of epigenetic mechanisms that might lead to the differential regulation of CAMs. Analysis of differential methylation using TCGA methylation data uncovered that out of the 181 deregulated CAMs, 32 CAMs showed differential methylation in GBM compared to control. We identified that 12 genes are upregulated corresponding to the 18 hypomethylated CpGs and 20 genes are downregulated corresponding to 28 hypermethylated CpGs **(Supplementary Fig.** **3****B, Supplementary Table** **4****)**. This was also validated in our patient cohort (GSE79122) and GSE60274 datasets, wherein most of the genes were similarly differentially methylated **(Supplementary Fig.** **3****C)**. Thus, we identified 6.6% of upregulated CAMs to be hypomethylated and 11% of downregulated CAMs to be hypermethylated.

It is well established that the gene transcript levels can be regulated by miRNAs. We investigated to identify the downregulated miRNAs predicted to target upregulated CAMs, and upregulated miRNAs predicted to target downregulated CAMs. We used TCGA expression data for both miRNA and mRNA, and identified nine downregulated miRNAs that can putatively target 52 upregulated CAMs, and five upregulated miRNAs can putatively target 43 downregulated CAMs in GBM **(Supplementary Fig.** **3****D, Supplementary Table** **5****)**. Thus, 59% of the upregulated CAMs and 46.2% of the downregulated CAMs were regulated by miRNAs suggesting that miRNAs play a pivotal role in regulating differentially expressed CAMs apart from copy number variation and DNA methylation **(Supplementary Fig.** **3****E)**.

### Prostaglandin F2 receptor inhibitor (PTGFRN) is upregulated in GBM and is a poor prognostic indicator

Towards predicting the prognostic values of CAMs, we carried out Univariate Cox regression analysis using the TCGA dataset. This analysis identified 127 CAMs to be significantly correlated with survival. Among them, 84 CAMs were found to correlate to poor prognosis, and 43 CAMs were found to correlate to good prognosis in GBM **(Supplementary Table** **6****)**. A transmembrane scaffolding protein PTGFRN, which predicted poor prognosis in GBM, was chosen for in-depth investigation due to various following reasons. First, patients with high PTGFRN transcripts had significantly lower median survival than those with low PTGFRN transcripts **(**Fig. 1B**)**. Second, PTGFRN transcript levels were significantly upregulated in GBM compared to control brain samples in multiple datasets **(**Fig. 1C**)**. Third, it was observed that PTGFRN expression was found to be significantly upregulated: in classical and mesenchymal subtypes as compared to neural and pro-neural subtypes **(**Fig. 1D**)**, in wild type IDH1 tumors as compared to mutant IDH1 tumors, and in G-CIMP– tumors as compared to G-CIMP+ tumors of GBM **(**Fig. 1E**)**. We also observed that most of the samples were positive for PTGFRN protein in IHC studies in GBM **(**Fig. 1F, G**)**. Fourth, it was found to be significantly upregulated in GSCs compared to corresponding DGCs (see in Fig. 4). It is reported that wild-type IDH1 and G-CIMP– subtypes of GBM are more aggressive than their counterparts [24]. However, no significant difference in the expression of PTGFRN was found between MGMT methylated and unmethylated groups of GBM **(**Fig. 1E**)**. Further, the transcript level of PTGFRN was upregulated in most of the GBM cell lines compared to control brain tissues and SVG **(**Fig. 1H**)**. The protein levels were varying across the cell lines but distinctly higher in U373, T98G, A172, and LN229, compared to SVG. However, we observed that PTGFRN transcript and protein levels in IHA were at similar levels as that of GBM cell lines **(**Fig. 1I**)**. Thus, the results so far indicate that the PTGFRN is highly upregulated in GBM, associated with wild type IDH1 and G-CIMP– GBMs, and is a predictor of poor prognosis in GBM.

### PTGFRN regulates cell growth, migration, and invasion in GBM

**Fig. 2:**
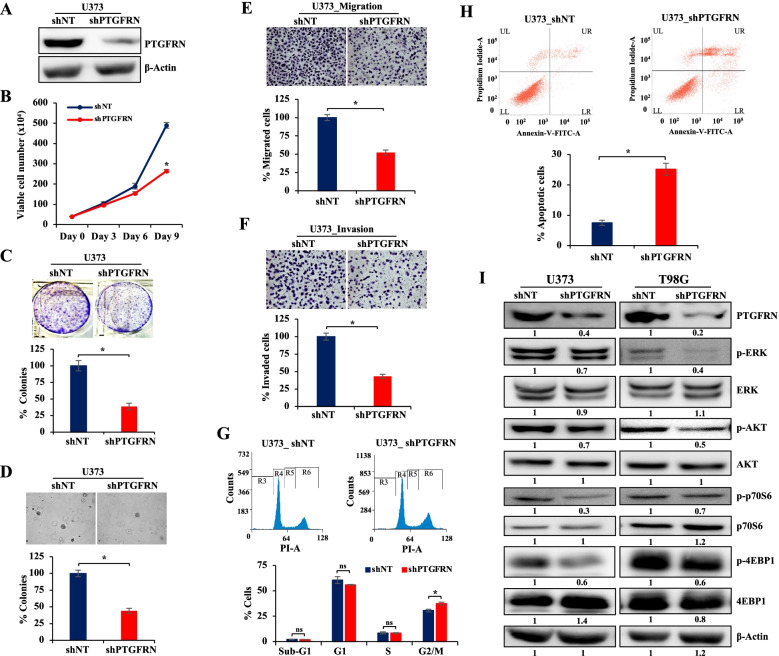
Knockdown of PTGFRN diminishes cell growth, migration, and invasion in GBM. In U373 cells PTGFRN silenced with either shNT or shPTGFRN, **A** immunoblot shows protein levels of PTGFRN and β-Actin served as a loading control (required portion of the blot is shown after cropping from the whole blot for both the proteins), **B** line graph shows the relative cell viability. Representative images show **(C)** colony number, **(D)** soft agar colony number, **(E)** migration, and **(F)** invasion after silencing PTGFRN in U373, and quantification is shown as bar graphs. **(G)** Histograms represent the DNA content (stained with PI) in control and PTGFRN silenced cells in U373 and the bar graph represents the quantification of percentage of cells in different phases of cell cycle. **(H)** Flow cytometry dot plots represent the Annexin-V positive cells in control and PTGFRN silenced cells in U373 and quantification showed as a bar graph, for quantification UR and LR regions of the plot were considered. **(I)** Immunoblots show the protein levels of PTGFRN, p-ERK, ERK, p-AKT, AKT, p-p70S6, p70S6, p-4EBP1, and 4EBP1 after silencing PTGFRN in U373 and T98G. β-Actin is used as a loading control in western blotting (the required portion of the blot is shown after cropping from the whole blot for all the proteins). The quantification for each blot is given below the blot. The significance was tested using the Student’s t-test and the symbols are indicated as follows: (ns) not significant; (*) *p ≤* 0.05; (**) *p ≤* 0.01 and (***) *p ≤* 0.001

To explore the role of PTGFRN in glioma development, silencing studies were carried out in different glioma cell lines. We chose glioma cell lines based on different PTGFRN expression levels, high (T98G), moderate (U373 and U251), and low (U87), for further studies. PTGFRN was silenced using specific short hairpin RNA (shRNA), and the silencing was verified by western blot **(**Fig. 2A**, Supplementary Fig.** **4****A)**. Silencing PTGFRN significantly reduced cell proliferation, colony formation, anchorage-independent growth, migration, and invasion in U373 **(**Fig. 2B-F**)**, U343, T98G, U251, and U87 glioma cells **(Supplementary Fig.** **4****B-****4****F)**. Silencing PTGFRN caused G2/M arrest in U373, whereas G1 arrest in T98G cells **(**Fig. 2G**, Supplementary Fig.** **4****G)**. Further, we found that silencing PTGFRN significantly increased Annexin-V positive cells in U373 and U251 by 18 and 10% of the total cells, respectively **(**Fig. 2H**, Supplementary Fig.** **4****H)**. We also investigated the role of PTGFRN in the regulation of signaling pathways that are highly dysregulated in GBM, such as ERK, AKT, and mTOR. Silencing of PTGFRN in U373 and T98G reduced phospho-AKT, phospho-ERK, phospho-4EBP1, and phospho-p70S6 **(**Fig. 2I**)**. These results collectively suggest that PTGFRN could be playing an essential role in growth, migration, and invasion in GBM.

### PTGFRN expression is regulated by promoter DNA methylation and miR-137 in GBM

**Fig. 3:**
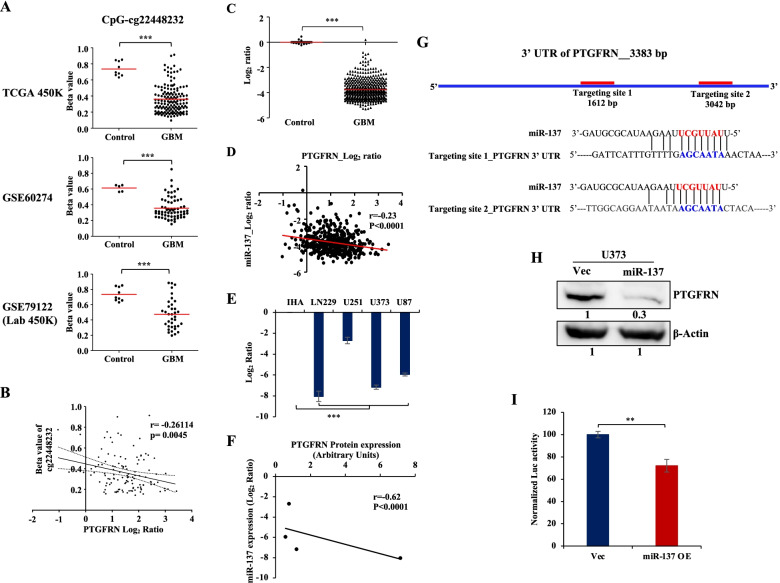
Regulation of PTGFRN by promoter methylation and miR-137. **A** Scatter plots depicting the beta values for CpGs cg22448232 in control and GBM samples in TCGA 450 K, GSE60274, and GSE79122 datasets. **B** The correlation graph shows the correlation between CpG methylation of cg22448232 and the expression of PTGFRN in TCGA GBM samples. **C** Scatter plot depicting the transcript levels of miR-137 in control and GBM samples in TCGA dataset. **D** Dot plot represents the correlation between expression of PTGFRN and miR-137 in TCGA GBM samples. **E** The bar graph shows the transcript levels of miR-137 in IHA and GBM cell lines. **F** The correlation graph shows the correlation between protein levels of PTGFRN (normalized values from Fig. 1I) and the miR-137 levels (log_2_ ratio values from Fig. 3E) in GBM cell lines. **G** Schematic shows miR-137 targeting sites on the 3’UTR of PTGFRN and base pairing between miR-137 and targeted sequence in the 3’UTR of PTGFRN. **(H)** Immunoblot shows PTGFRN protein levels in vector and miR-137 overexpression in U373 and β-Actin was used as a loading control (required portion of the blot is shown after cropping from the whole blot for both the proteins). **I** The bar graph is depicting the normalized luciferase activity of pmiR-GLO-3’UTR of PTGFRN in pcDNA3.2/V5-Vector and pcDNA3.2/V5-miR-137 overexpression conditions. The significance was performed using the Wilcoxon-Mann-Whitney test or Student's t-test and the symbols are indicated as follows: (ns) not significant; (*) *p ≤* 0.05; (**) *p ≤* 0.01 and (***) *p ≤* 0.001

The analysis of mechanisms behind the deregulation of CAMs in GBM revealed that PTGFRN might be regulated by DNA CpG methylation and miRNAs. DNA methylation analysis of PTGFRN promoter found that the CpG probe cg2248232 to be hypomethylated significantly in GBM compared to control in TCGA 450 K methylation array, GSE60274 (27 K array), and GSE79122 (450 K array) methylation datasets **(**Fig. 3A**)**. We performed a correlation analysis between PTGFRN transcript level and CpG methylation in GBM and found a significant negative correlation between expression and methylation **(**Fig. 3B**)**. Further, miRNA predicting algorithms in miRwalk (http://zmf.umm.uniheidelberg.de/apps/-zmf/mirwalk2/) predicted that miR-137, miR-107, and miR-133b could target 3’UTR of PTGFRN. We also found that these three miRs were significantly downregulated in GBM compared to control in TCGA dataset **(Supplementary Table** **5****,** Fig. 3C**, Supplementary Fig.** **5****A, B)**. We performed a correlation analysis in which we found a significant negative correlation between the expression of PTGFRN and miR-137 in GBM **(**Fig. 3D**)**. However, no significant correlation was found between the expression of PTGFRN and either miR-107 or miR-133b in GBM (**Supplementary Fig.** **5****C, D**). Next, we checked the transcript levels of miR-137 in IHA and GBM cell lines and found that miR-137 levels to be significantly downregulated in GBM cell lines as compared to IHA **(**Fig. 3E**)**. We also found a significant negative correlation between the levels of miR-137 and the protein levels of PTGFRN in GBM cell lines **(**Fig. 3F**)**. It was observed that miR-137 putatively targets two specific sites in 3’UTR of PTGFRN **(**Fig. 3G**)**. The overexpression of miR-137 dramatically reduced the protein levels of PTGFRN in U373 **(**Fig. 3H**)**. Further, to validate the negative effect of miR-137 on PTGFRN expression we performed 3’UTR luciferase reporter assay. We co-transfected either pcDNA3.2/V5 vector or pcDNA3.2/V5-miR-137 overexpression plasmid along with the pmiR-GLO-3’UTR of PTGFRN plasmid and found a significant reduction in luciferase activity in miR-137 overexpressed condition as compared to the vector control **(**Fig. 3I**)**. From these results, we conclude that the promoter hypomethylation and miR-137 downregulation might be responsible for the upregulation of PTGFRN in GBM.

### PTGFRN is upregulated in glioma stem-like cells (GSCs) and is essential for GSC growth

**Fig. 4:**
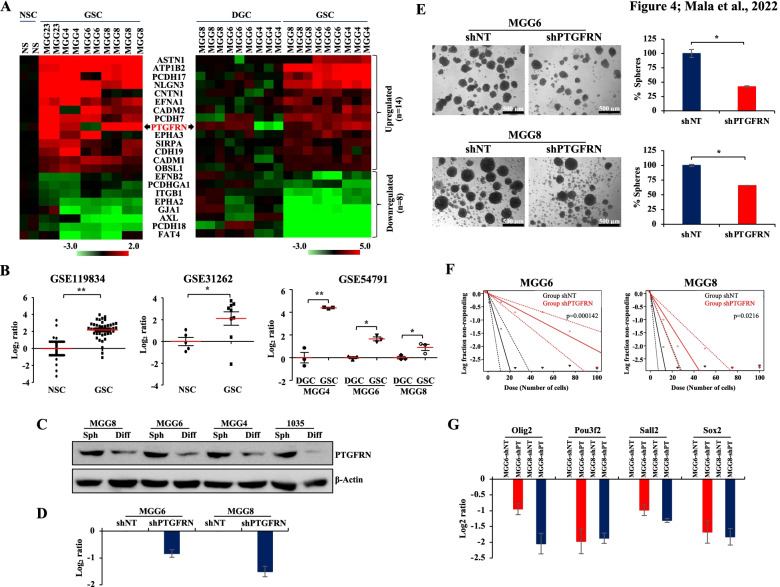
PTGFRN is upregulated in GSCs and required for its growth. **A** Heatmaps representing the differentially expressed CAMs in GSCs as compared to NSCs (left) and in GSCs as compared to DGCs (right). GSE46016 (GSC vs NSC, gene microarray) and GSE54791 (GSC vs DGC, RNA-Seq) datasets were used for the analysis. Red and green color indicates the upregulated and downregulated CAMs, respectively. **B** Scatter plots represent the transcript levels of PTGFRN in different datasets GSE119834, GSE31262, and GSE54791. **C** Immunoblot shows the protein levels of PTGFRN in GSCs and corresponding DGCs in MGG8, MGG6, MGG4, and 1035 and β-Actin was used as a loading control (required portion of the blot is shown after cropping from the whole blot for both the proteins). GSCs cultured as neurospheres indicated as Sph (Spheroid culture) and DGCs cultured as monolayer and indicated as Diff (Differentiated cells). **D** The bar graph shows the transcript levels of PTGFRN in shNT and shPTGFRN in MGG6 and MGG8. **E** Representative images of neurospheres and their quantification in MGG6 and MGG8 after silencing PTGFRN. **F** Line graphs show the limiting dilution analysis in MGG6 and MGG8 after silencing PTGFRN. **G** Bar diagram shows the transcript levels of Olig2, Pou3f2, Sall2, and Sox2 in shNT and shPTGFRN (represented as shPT) in MGG6 and MGG8. The significance was performed using the Wilcoxon-Mann-Whitney test or Student’s t-test and the symbols are indicated as follows: (ns) not significant; (*) *p ≤* 0.05; (**) *p ≤* 0.01 and (***) *p ≤* 0.001

To dissect the role of CAMs in GSCs, we investigated the transcriptome profile of GSCs and their corresponding differentiated glioma cells (DGCs) and neural stem cells (NSCs). The trasncriptome data for GSCs versus DGCs and GSCs versus NSCs was obtained from GSE54791 and GSE46016 datasets, respectively. Bioinformatics analysis revealed that 14 CAMs were significantly upregulated, and 8 CAMs were significantly downregulated specifically in GSCs compared to both NSCs and DGCs **(**Fig. 4A**)**. Since we found PTGFRN to be one of the most upregulated CAMs in GSCs, we analyzed its expression in various GSC datasets. We found that PTGFRN transcript levels to be significantly upregulated in GSCs compared to NSCs (GSE119834, GSE31262) and DGCs (GSE54791) **(**Fig. 4B**)**. Further, we also observed that PTGFRN protein levels were more in MGG8, MGG6, MGG4, and 1035 GSCs than their corresponding DGCs **(**Fig. 4C**)**. Silencing PTGFRN in MGG6, MGG8, and U343 significantly reduced neurosphere formation as assessed by neurosphere assay and limiting dilution analysis **(**Fig. 4D-F**, Supplementary Fig.** **5****E, F)**. Further, we analyzed the transcript levels of GSC reprogramming factors, Olig2, Pou3f2, Sall2, and Sox2 [25] in MGG6 and MGG8 after silencing of PTGFRN. A decrease in the expression of Olig2, Pou3f2, Sall2, and Sox2 in MGG6 and MGG8 was found after silencing of PTGFRN compared to shNT (Fig. 4G). From these results, we conclude that PTGFRN is upregulated in GSCs and is essential for GSC growth.

## Discussion

Alteration in CAMs is one of the main reasons for enhanced migration and invasion in GBM [26]. In this study, we identified nearly 35% of CAMs analyzed were found to be differentially regulated in GBM. Our results corroborate previous reports that CAMs exhibit both oncogenic and tumor suppressor functions in GBM [27, 28]. Further, we explored the mechanism behind the differential expression of CAMs in GBM. More than 50% of deregulated CAMs were identified to be targeted by miRNA, indicating their critical role in the deregulation of CAMs. Some of these miRNA-mRNA pairs have already been reported to play a role in glioma biology [29].

We found that PTGFRN expression is high in glioma tissues, cell lines, and GSCs, and its increased expression correlates with poor patient survival, corroborating previous reports [8, 21, 30]. We observed that PTGFRN is required for cell growth, migration, invasion, progression of the cell cycle, and evading apoptosis in glioma cells. These results suggest its oncogenic role in glioma. It was reported that PTGFRN plays an essential role in muscle regeneration [17] and cell migration [15]. Hence, its high expression could protect cells from apoptosis, thereby promoting growth and migration in GBM. Abnormal activation of PI3K/AKT/mTOR signaling has been implicated in tumor initiation, progression, and therapeutic resistance in glioma [31]. We found that silencing PTGFRN reduced ERK, AKT, and mTOR signaling in glioma cell lines. This suggests that PTGFRN could be functioning through transmembrane receptor signaling that modulates pro-survival and promigratory signaling pathways in cancer [32]. Our results were further strengthened by the recent reports wherein PTGFRN was found to be conferring resistance to radiation via PI3K-AKT signaling in GBM [21] and its expression correlated with metastatic capacity in lung cancer [7]. We observed that silencing of PTGFRN leads to G2/M arrest in U373 and G1 arrest in T98G cells. While we do not know the reason behind the different impacts between two cell lines, this could be due to unknown genetic changes unique to each cell line. In a recent report, cell cycle defects were seen in PTGFRN silenced neurospheres [21]. We identified PTGFRN expression could be regulated by promoter hypomethylation and downregulation of miR-137, which is predicted to target PTGFRN in GBM. Our results concur with the findings of the previous report concerning miR-137 [33]. PTGFRN is essential for the bioactivity of extracellular vesicles released by perivascular stem cells which in turn stimulate bone repair and also confer resistance to radiation in GSCs [21, 34]. In our study, silencing PTGFRN inhibited GSC growth and survival associated with a decrease in levels of reprogramming factors, Pou3f2, Olig2, Sall2, and Sox2. These factors were reported to reprogram DGCs into GSCs and also maintain their stemness [25]. Our study thus emphasizes the role of PTGFRN in the stemness maintenance of GSCs.

## Conclusion

This study offers a comprehensive overview of the deregulation of CAMs and probable reasons for their regulation in GBM. This study also uncovers for the first time the promigratory role and regulation of PTGFRN in GBM apart from its role in cell growth. Studying the role of CAMs in GBM biology may be useful for targeting tumors, and PTGFRN can be used as a therapeutic target to treat invasive GBM.

## Supplementary Information

**Additional file 1. Figure S1. **Flow chart describing the use of various datasets to identify the importance of CAMs for glioma development and progression. CAMs (*n* = 518) derived from various sources that included CAMs family genes based on protein domain structures such as cadherin, integrin, and immunoglobulin and Gene Ontology (GO) terms related to cell adhesion and ‘CAMs and Homo sapiens’ keyword query against NCBI Entrez annotations and various literature, manually curated and compiled and were used in this study. The first and second branch depicts the use of TCGA Agilent dataset to identify differentially expressed CAMs in GBM followed by the possible causes of their differential regulation and survival analysis, respectively. The last branch identifies the GSC-specific CAMs over both NSCs and DGCs. Functional studies were carried out for PTGFRN which is deregulated in GBM and GSCs and indicates a poor prognosis. The number in brackets shows the number of samples used for analysis. GSCs: Glioma-like stem cells, NSCs: Normal neural stem cells, DGCs: Differentiated glioma stem cells, CNV: Copy number variation.

**Additional file 2. Figure S2. **Differentially regulated CAMs in GBM. (**A, B, and C**) Heatmaps indicating differentially regulated CAMs in REMBRANDT, GSE22866, and GSE7696, respectively.

**Additional file 3. Figure S3. **Role of CNV, methylation, and miRNA in the regulation of CAMs in GBM. (**A**) Waterfall plot depicting the copy number variation in CAMs altered in more than 1.5% samples of GBM. Each vertical line represents one sample. Red denotes amplification and blue denotes deletion of the CAMs. The number represents the proportion of the samples in which the CAM is amplified or deleted. (**B**) Heatmaps are representing the differentially regulated CAMs which are also differentially methylated in GBM in TCGA dataset. The yellow color indicates the hypermethylated CpGs (*n* = 18) which corresponds to downregulated genes (*n* = 20) shown in green, right. The blue color depicts the hypomethylated CpGs (*n* = 28) which corresponds to upregulated genes (*n* = 12) shown in red, right. (**C**) Heatmaps are representing the differentially methylated CpGs in GBM as compared to control in GSE7912 and GSE60274 datasets. The blue and yellow colors indicate hypomethylation and hypermethylation, respectively. (**D**) Tabular illustration represents the CAMs and the putative targeting miRNAs. Differentially expressed miRNAs predicted to target the CAMs were identified using miRwalk. Only those miRNAs which were predicted to target the CAMs in seven or more than seven algorithms in miRwalk and having reciprocal regulation as compared to targeted CAMs are shown. The green or red box indicates the predicted miRNA-CAM targeting pair, whereas the empty box indicates the non-targeting miRNA-CAM pair. Left: upregulated CAMs predicted to be targeted by downregulated miRNAs; right: downregulated CAMs predicted to be targeted by upregulated miRNAs. (**E**) Venn diagrams depicting the summary of CAMs regulation, top: the number of upregulated CAMs regulated by the 3 factors individually and in combination, bottom: the number of downregulated genes regulated by the 3 factors individually and in combination.

**Additional file 4. Figure S4. **Knockdown of PTGFRN reduces cell growth, migration, and invasion in GBM. (**A**) Western blot represents the protein levels of PTGFRN after silencing PTGFRN with either shPTGFRN or shNT in U343, T98G, U251, and U87 and β-Actin was used as a loading control (required portion of the blot is shown after cropping from the whole blot). After silencing PTGFRN with shPTGFRN or control shNT, (**B**) line graphs show the relative cell viability in U87, U343, T98G, and U251, (**C**) representative images of colonies in U343 and T98G, (**D**) images show the relative soft agar colonies in U343 and U251, (**E**) representative images of migration, and (**F**) invasion in U251 and the quantification showed as bar graphs. **(G)** Histograms represent the DNA content by PI staining to assess Cell cycle in T98G after silencing of PTGFRN and the bar graph represents the percentage of cells in different phases of the cell cycle. **(H)** Flow cytometry dot plots represent the annexin-V positive cell population in U251 after silencing PTGFRN and quantification showed as bar diagrams, for quantification UR and LR regions of the plot were considered. The Student’s t-test was performed to test the statistical significance and the symbols are indicated as follows: (ns) not significant; (*) *p* ≤ 0.05; (**) *p* ≤ 0.01 and (***) *p* ≤ 0.001.

**Additional file 5. Figure S5.** PTGFRN regulation by miRs and its importance in GSC survival. (**A**) Scatterplot represents the transcript levels of miR-107 in control and GBM in TCGA dataset. (**B**) The correlation graph shows the correlation between the expression of PTGFRN and miR-107 in TCGA GBM samples. (**C**) Scatter plot depicting the transcript levels of miR-133b in control and GBM samples in TCGA dataset. (**D**) The correlation graph shows the correlation between the expression of PTGFRN and miR-133b in TCGA GBM samples. (**E**) The bar graph shows the transcript levels of PTGFRN after silencing of PTGFRN either with shPTGFRN or shNT in U343 and (**F**) the representative images of neurospheres and their quantification. The Student’s t-test was performed to test the statistical significance and the symbols are indicated as follows: (ns) not significant; (*) *p* ≤ 0.05; (**) *p* ≤ 0.01 and (***) *p* ≤ 0.001.

**Additional file 6. Table S1.** List of CAMs catalogued and used in this study. **Table S2.** Differentially regulated CAMs in different datasets. **Table S3.** Differentially expressed CAMs which showed significant CNVs. **Table S4.** Differentially expressed CAMs which showed differetial methylation status. **Table S5**. Differentially expressed CAMs and the miRNAs predicted to target them. **Table S6.** Univariate cox regression.

## Data Availability

All the analyzed data are included as supplementary files. Materials and other data are available with the first author and corresponding author and will be furnished upon request. We utilized in whole or part of the data from The Cancer Genome Atlas pilot project funded by the NHGRI and NCI. The information about TCGA institutions and investigators that constitute TCGA network can be found at https://cancergenome.nih.gov. We also used REMBRANDT, GSE22866, GSE7696, GSE60274, GSE79122, GSE54791, GSE119834, GSE31262, and GSE46016 datasets in this study.

## Abbreviations

GBM: Glioblastoma
CAMs: Cell Adhesion Molecules
PTGFRN: Prostaglandin F2 receptor inhibitor
TCGA: The Cancer Genome Atlas
IHA: Immortalized human astrocytes
GSC: Glioma stem-like cell
DGC: Differentiated glioma cell
NSC: Neural stem cell
Ig: Immunoglobulin
ECM: Extracellular matrix
miR: Micro-RNA
