# Supplementary figures and images for: Integrative analysis of cell adhesion molecules in glioblastoma identified prostaglandin F2 receptor inhibitor (PTGFRN) as an essential gene
Source: BMC Cancer. 2022 Jun 11;22:642. doi: 10.1186/s12885-022-09682-2 (PMC9188228; doi:10.1186/s12885-022-09682-2)

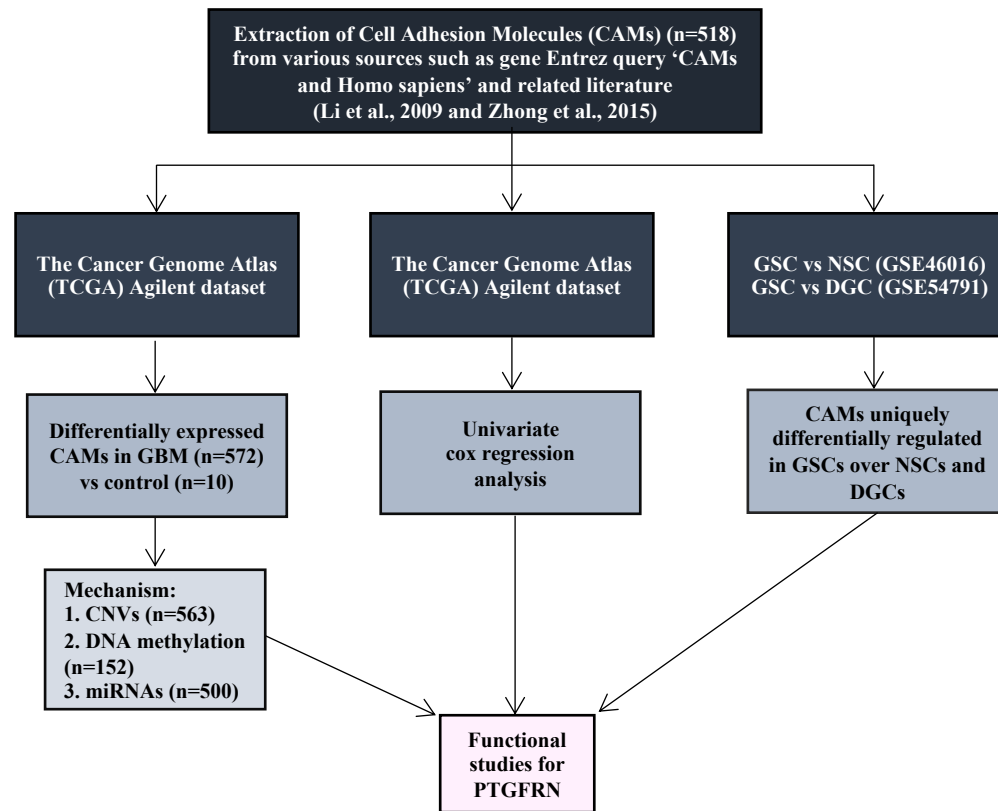

Supplement: Supplementary file 1 — Additional file 1. Figure S1. Flow chart describing the use of various datasets to identify the importance of CAMs for glioma development and progression. CAMs (n = 518) derived from various sources that included CAMs family genes based on protein domain structures such as cadherin, integrin, and immunoglobulin and Gene Ontology (GO) terms related to cell adhesion and ‘CAMs and Homo sapiens’ keyword query against NCBI Entrez annotations and various literature, manually curated and compiled and were used in this study. The first and second branch depicts the use of TCGA Agilent dataset to identify differentially expressed CAMs in GBM followed by the possible causes of their differential regulation and survival analysis, respectively. The last branch identifies the GSC-specific CAMs over both NSCs and DGCs. Functional studies were carried out for PTGFRN which is deregulated in GBM and GSCs and indicates a poor prognosis. The number in brackets shows the number of samples used for analysis. GSCs: Glioma-like stem cells, NSCs: Normal neural stem cells, DGCs: Differentiated glioma stem cells, CNV: Copy number variation. [file 12885_2022_9682_MOESM1_ESM.pdf]

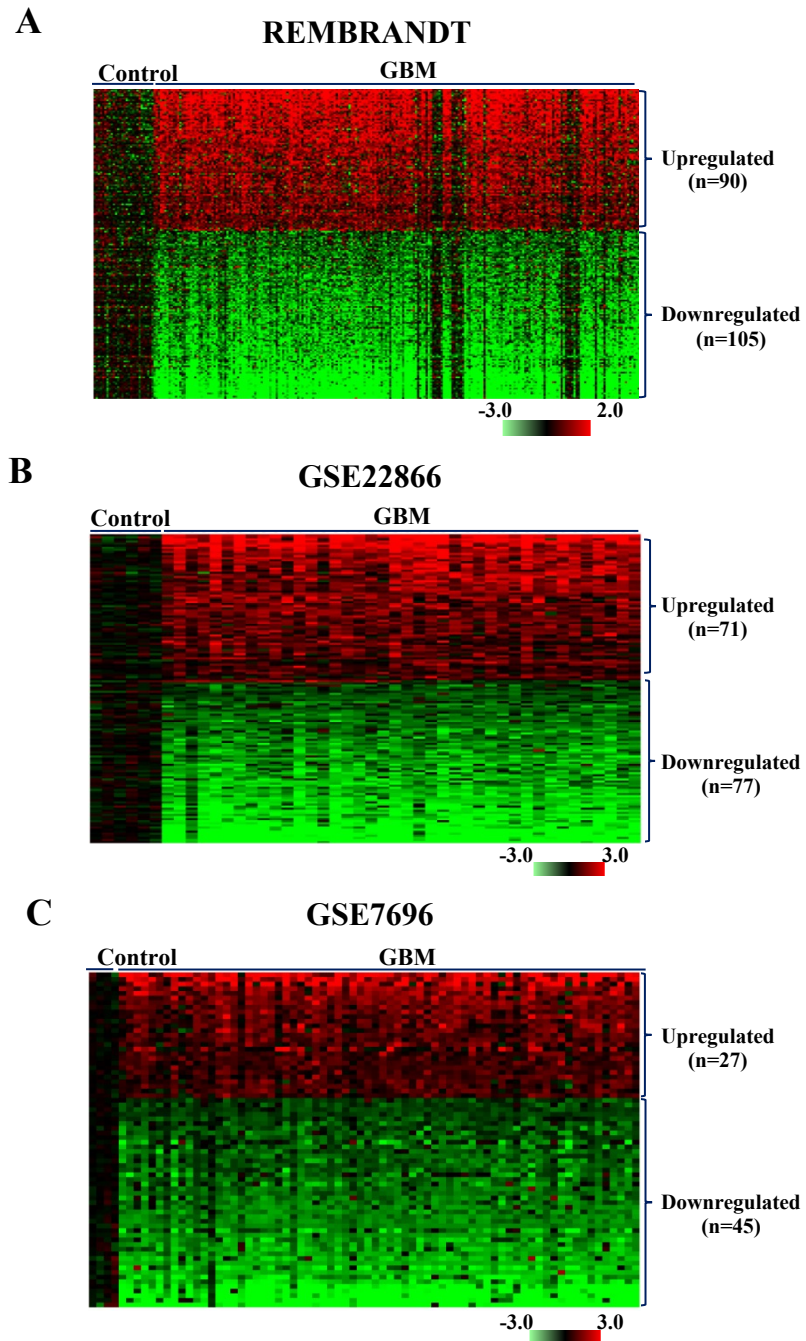

Supplement: Supplementary file 2 — Additional file 2. Figure S2. Differentially regulated CAMs in GBM. (A, B, and C) Heatmaps indicating differentially regulated CAMs in REMBRANDT, GSE22866, and GSE7696, respectively. [file 12885_2022_9682_MOESM2_ESM.pdf]

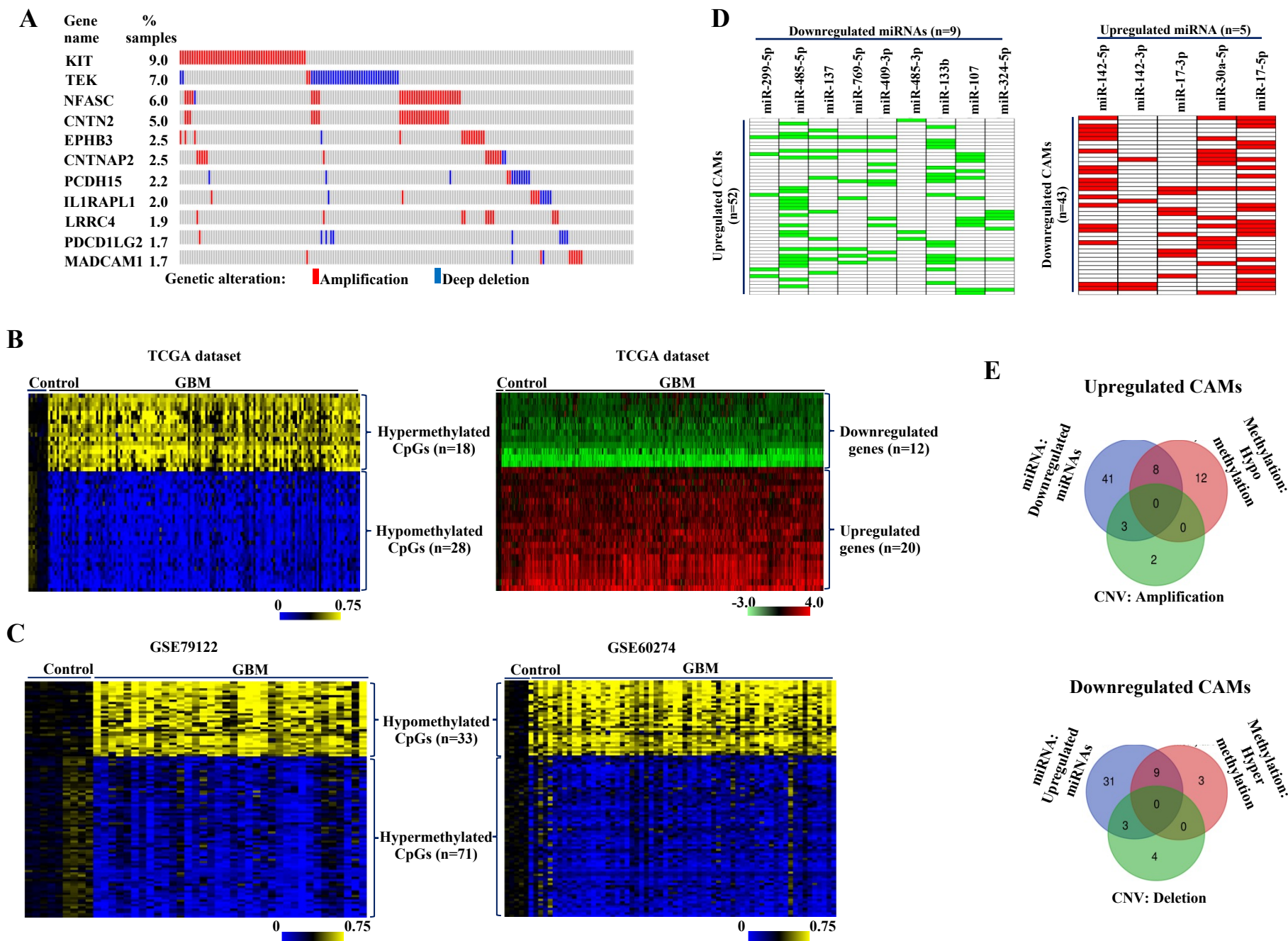

Supplement: Supplementary file 3 — Additional file 3. Figure S3. Role of CNV, methylation, and miRNA in the regulation of CAMs in GBM. (A) Waterfall plot depicting the copy number variation in CAMs altered in more than 1.5% samples of GBM. Each vertical line represents one sample. Red denotes amplification and blue denotes deletion of the CAMs. The number represents the proportion of the samples in which the CAM is amplified or deleted. (B) Heatmaps are representing the differentially regulated CAMs which are also differentially methylated in GBM in TCGA dataset. The yellow color indicates the hypermethylated CpGs (n = 18) which corresponds to downregulated genes (n = 20) shown in green, right. The blue color depicts the hypomethylated CpGs (n = 28) which corresponds to upregulated genes (n = 12) shown in red, right. (C) Heatmaps are representing the differentially methylated CpGs in GBM as compared to control in GSE7912 and GSE60274 datasets. The blue and yellow colors indicate hypomethylation and hypermethylation, respectively. (D) Tabular illustration represents the CAMs and the putative targeting miRNAs. Differentially expressed miRNAs predicted to target the CAMs were identified using miRwalk. Only those miRNAs which were predicted to target the CAMs in seven or more than seven algorithms in miRwalk and having reciprocal regulation as compared to targeted CAMs are shown. The green or red box indicates the predicted miRNA-CAM targeting pair, whereas the empty box indicates the non-targeting miRNA-CAM pair. Left: upregulated CAMs predicted to be targeted by downregulated miRNAs; right: downregulated CAMs predicted to be targeted by upregulated miRNAs. (E) Venn diagrams depicting the summary of CAMs regulation, top: the number of upregulated CAMs regulated by the 3 factors individually and in combination, bottom: the number of downregulated genes regulated by the 3 factors individually and in combination. [file 12885_2022_9682_MOESM3_ESM.pdf]

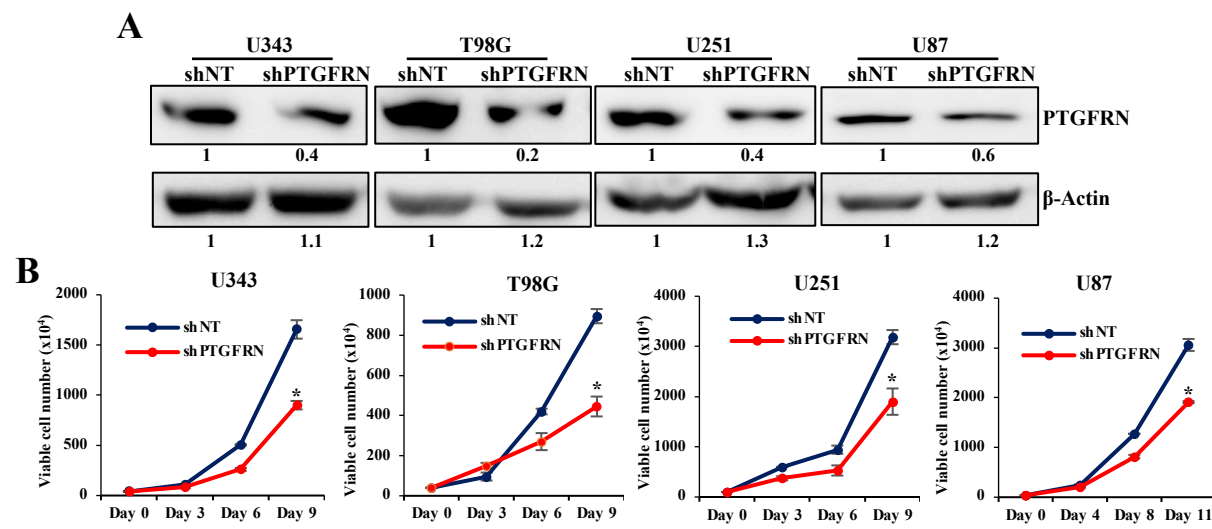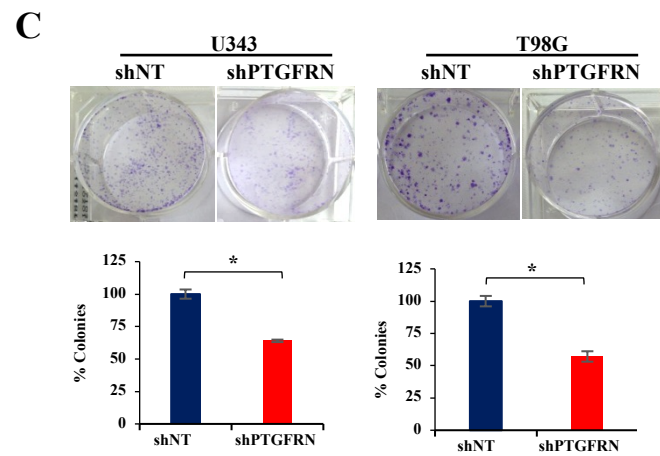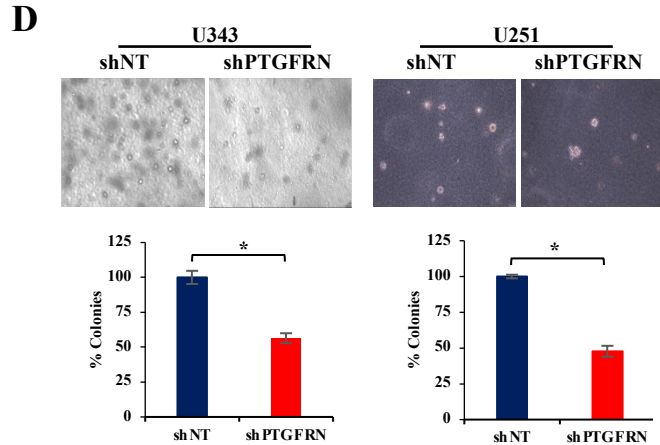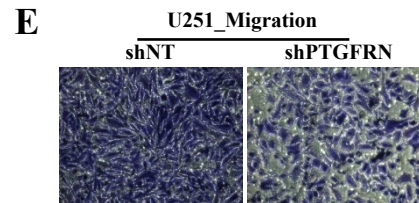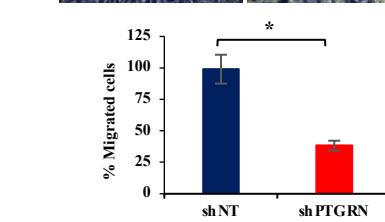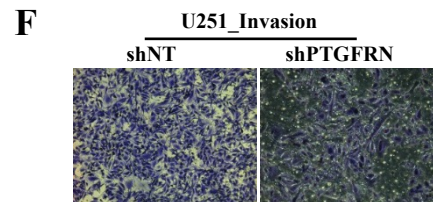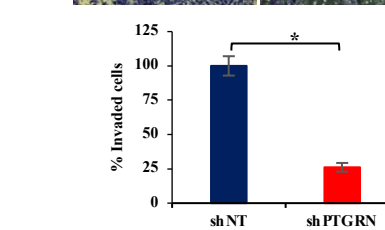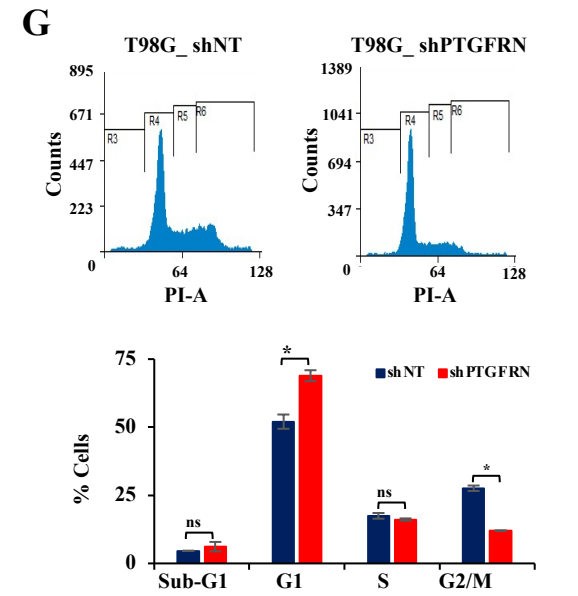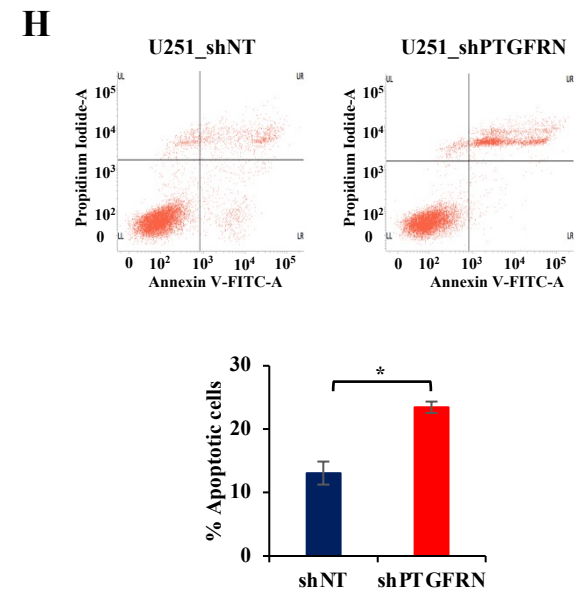

Supplement: Supplementary file 4 — Additional file 4. Figure S4. Knockdown of PTGFRN reduces cell growth, migration, and invasion in GBM. (A) Western blot represents the protein levels of PTGFRN after silencing PTGFRN with either shPTGFRN or shNT in U343, T98G, U251, and U87 and β-Actin was used as a loading control (required portion of the blot is shown after cropping from the whole blot). After silencing PTGFRN with shPTGFRN or control shNT, (B) line graphs show the relative cell viability in U87, U343, T98G, and U251, (C) representative images of colonies in U343 and T98G, (D) images show the relative soft agar colonies in U343 and U251, (E) representative images of migration, and (F) invasion in U251 and the quantification showed as bar graphs. (G) Histograms represent the DNA content by PI staining to assess Cell cycle in T98G after silencing of PTGFRN and the bar graph represents the percentage of cells in different phases of the cell cycle. (H) Flow cytometry dot plots represent the annexin-V positive cell population in U251 after silencing PTGFRN and quantification showed as bar diagrams, for quantification UR and LR regions of the plot were considered. The Student’s t-test was performed to test the statistical significance and the symbols are indicated as follows: (ns) not significant; (*) p ≤ 0.05; (**) p ≤ 0.01 and (***) p ≤ 0.001. [file 12885_2022_9682_MOESM4_ESM.pdf]

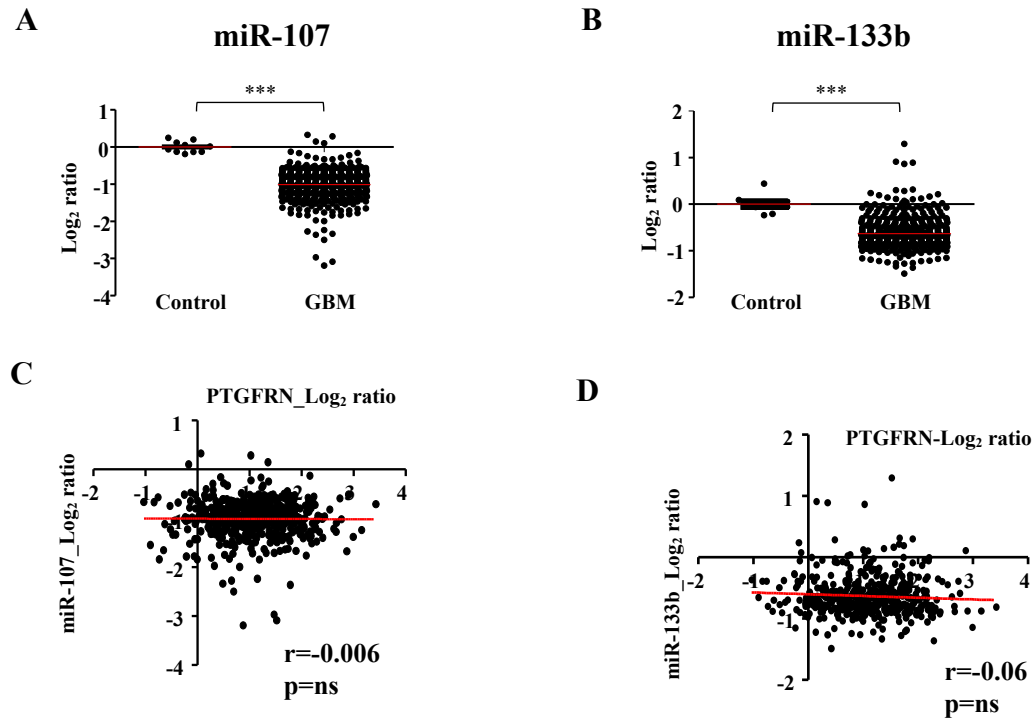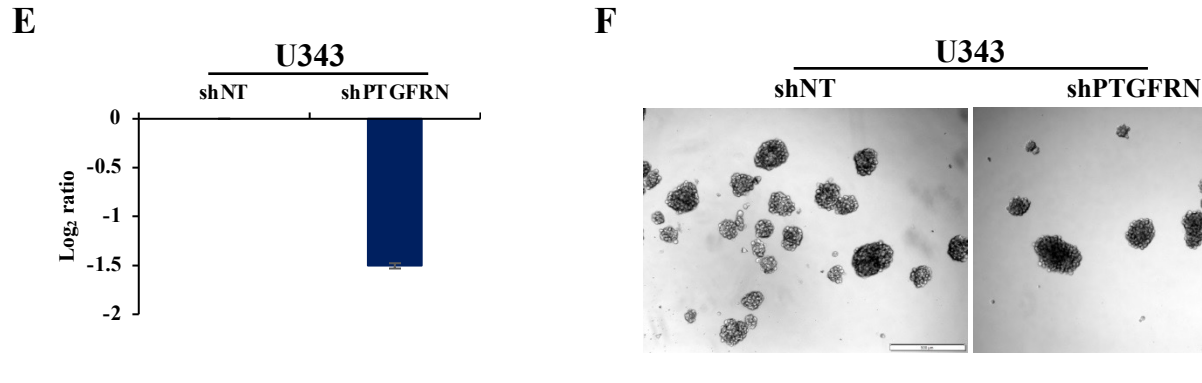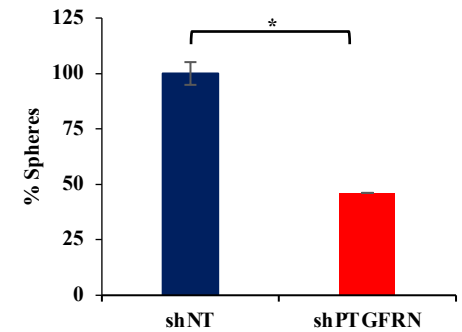

Supplement: Supplementary file 5 — Additional file 5. Figure S5. PTGFRN regulation by miRs and its importance in GSC survival. (A) Scatterplot represents the transcript levels of miR-107 in control and GBM in TCGA dataset. (B) The correlation graph shows the correlation between the expression of PTGFRN and miR-107 in TCGA GBM samples. (C) Scatter plot depicting the transcript levels of miR-133b in control and GBM samples in TCGA dataset. (D) The correlation graph shows the correlation between the expression of PTGFRN and miR-133b in TCGA GBM samples. (E) The bar graph shows the transcript levels of PTGFRN after silencing of PTGFRN either with shPTGFRN or shNT in U343 and (F) the representative images of neurospheres and their quantification. The Student’s t-test was performed to test the statistical significance and the symbols are indicated as follows: (ns) not significant; (*) p ≤ 0.05; (**) p ≤ 0.01 and (***) p ≤ 0.001. [file 12885_2022_9682_MOESM5_ESM.pdf]
